# A Meta-Analysis of Gray Matter Differences Between Bilinguals and Monolinguals

**DOI:** 10.3389/fnhum.2020.00146

**Published:** 2020-04-23

**Authors:** Anastasiya Danylkiv, Anthony J. Krafnick

**Affiliations:** Psychology Department, Dominican University, River Forest, IL, United States

**Keywords:** bilingualism, activation likelihood estimate analysis (ALE), voxel-based morphometry (VBM), gray matter, meta-analysis

## Abstract

Bilingualism is of great interest to the neuroscience of language, and understanding the anatomical changes associated with second language learning help inform theories of bilingual advantage across the lifespan. While the literature on structural differences between bilinguals and monolinguals is robust, relatively few studies of gray matter (GM) have directly compared bilinguals with monolinguals in a whole-brain analysis. Overall, this and heterogeneity of study samples and methodology have led to a lack of clear anatomical support for major theories. Here, we engage in an activation likelihood estimate (ALE) meta-analysis of voxel-based morphometry (VBM) studies of GM for cases that directly compare bilingual and monolingual subjects in a whole-brain analysis. The analysis (sixteen foci, from ten contrasts across eight studies) resulted in one cluster located primarily within the anterior lobe of the right cerebellum. However, when the one pediatric study was removed, the analysis revealed no consistent results across the studies included in this meta-analysis. This suggests that for VBM studies of bilingual and monolingual adults there is considerable heterogeneity of results that complicate the understanding of the bilingual brain. Future studies will need to include larger, more well-defined samples and interrogate more fine-grained anatomical features such as cortical thickness and surface area in order to more fully examine the anatomical changes associated with bilingualism across the lifespan.

## Introduction

Bilingualism continues to be a topic of intense interest, providing a unique lens into the study of the neuroscience of language. However, it also holds relevance more broadly in the realm of understanding how the brain is able to acquire a skill, taking advantage of language learning being a very common task. Benefits of learning multiple languages have been discussed and debated at length (Bialystok, 2017; Antoniou, 2019), and recent reviews have discussed topics including general cognitive benefits, enhanced neuroplasticity, and protection against aging (Baum and Titone, 2014; Li et al., 2014; Bialystok et al., 2016; Grundy et al., 2017). While there are different theories on how these benefits develop and manifest themselves (Green and Abutalebi, 2013; Abutalebi and Green, 2016; Grundy et al., 2017), the aforementioned reviews detail the evidence that suggest benefits to acquiring multiple languages exist. The proposed gains of learning a second language do come with some caveats, for example, age of acquisition appears to play a strong role in cognitive and brain changes associated with bilingualism (Berken et al., 2017) and immersion in the language being learned may also impact structural changes observed in the brain (Stein et al., 2014). Along with the importance of understanding the potential benefits of multi-language learning comes the concern of heterogeneity of samples in studies of bilingualism and the brain due to the roles of factors such as age of acquisition and how the second language is learned. As such Garcia-Penton et al. (2016), has outlined how the variability in sample selection and methodology have led to a lack of generalizability of results across studies, and relatively little neuroanatomical support for major theories of bilingualism. It is therefore important to understand where consistencies and inconsistencies exist across the literature of bilingualism and the brain, both for the current understanding of the neuroscience of language and learning and in planning future studies to adequately address the gaps in the existing knowledge base.

In terms of the impact of bilingualism on brain structure, a common tool for investigating differences in gray matter (GM) is voxel-based morphometry (VBM; Ashburner and Friston, 2000, 2005; Mechelli et al., 2005; Ashburner, 2007; Douaud et al., 2007). While there are concerns over whether this is the best measure for probing brain differences and relationships with neurocognitive measures (e.g., cortical thickness and surface area may provide more unique information; Panizzon et al., 2009; Winkler et al., 2010), it has been a very common tool used in neuroimaging studies of bilingualism. Overall, studies utilizing VBM in bilingualism have found increased GM in frontal, parietal, and cingulate regions associated with second language ability and acquisition, with motor system involvement including aspects of the basal ganglia (Grundy et al., 2017). However, while many studies utilize this technique, relatively few studies have directly compared bilingual and monolingual individuals in whole-brain analyses. For example, there have been investigations into simultaneous vs. sequential bilinguals (Kaiser et al., 2015; Berken et al., 2016), early vs. late bilinguals (Wei et al., 2015), high vs. low proficiency in second language (Reiterer et al., 2011), bilinguals vs. multilinguals (Grogan et al., 2012), correlational studies of gray matter volume with neurocognitive measures (e.g., Abutalebi et al., 2012; Martínez-Horta et al., 2019), and longitudinal studies of adults learning a new language (Osterhout et al., 2008; Stein et al., 2012; Deluca et al., 2018). Additionally, of the VBM studies that do directly compare monolinguals and bilinguals, several are region of interest (ROI) based and do not examine effects across the entire brain (Zou et al., 2012; Abutalebi et al., 2013, 2015; Del Maschio et al., 2018). While the approaches across these studies have provided important information in understanding the bilingual brain, in order to fully understand how the bilingual brain differs from the monolingual brain it is necessary to utilize whole-brain direct comparisons of the two groups.

At the time of writing, to our knowledge there have been ten experiments across eight studies that have employed a whole-brain comparison of gray matter between bilinguals and monolinguals using VBM (Mechelli et al., 2004; Ressel et al., 2012; Gold et al., 2013; Abutalebi et al., 2014, 2015; Pliatsikas et al., 2014; Olulade et al., 2016; García-Pentón et al., 2019). These studies vary considerably in several aspects of the sample characteristics, including first (L1) and second (L2) language, age of acquisition of L2 for the bilingual group, language proficiency, sample size, covariates of no interest included in the analyses, and in overall reporting of the sample details. There is also considerable variation in the methodology, such as multiple comparison correction and software package used (i.e., FSL^1^, Douaud et al., 2007; or SPM^2^, Ashburner and Friston, 2000). As previously discussed (Garcia-Penton et al., 2016), this variation also has likely contributed to an overall inconsistent literature and lack of clear evidence to back up theory.

The present study presents an activation likelihood estimate (ALE) meta-analysis of VBM studies of GM with direct whole-brain comparisons of bilinguals and monolinguals, in an effort to test the consistency of brain differences across these studies. Considering the variability in samples and details of the methodology (as described by Garcia-Penton et al., 2016) it was expected that there would be little consistency in the results, highlighting the need for larger and more well-defined studies to better understand the relationship between bilingualism and the brain.

## Methods

### Selection of Studies

We searched for articles on PubMed^3^ and Google Scholar (google.com/scholar) using the search terms: “voxel-based morphometry,” “gray matter volume,” “bilingualism,” “differences,” and “brain.” Additionally, reference lists from publications were inspected to discover additional relevant articles, and articles that cited eligible studies were reviewed using Google Scholar to maximize inclusion of relevant articles. For this meta-analysis, we only selected studies that used whole-brain VBM analyses to compare GM between bilingual and monolingual participants. Our inclusion criteria were as follows: (1) the study used VBM analysis (FSL-Douaud et al., 2007 or SPM-Ashburner and Friston, 2000); (2) both monolingual and bilingual subjects were included; (3) subjects were healthy and did not report neurological/psychiatric disorders; and (4) foci were generated from a whole-brain analysis. Our exclusion criteria were as follows: (1) data generated from ROI analyses; (2) subjects from patient populations that may impact neurological status; (3) non-VBM studies of volume; (4) group comparisons other than bilingual vs. monolingual (e.g., simultaneous vs. sequential bilinguals, bilinguals vs. multi-linguals); and (5) studies of bimodal bilinguals (e.g., English/American Sign-Language).

The last exclusion criteria for bimodal bilinguals is based on the different experience of these bilingual individuals (simultaneous vs. independent usage), and evidence that suggests cognitive control and brain related differences may not exist compared to monolingual individuals (Emmorey et al., 2008; Olulade et al., 2016). It is important to note that we did not exclude studies for age range of participants or L1/L2 language (other than bimodal bilinguals as noted above), as there are not enough studies fitting the criteria to investigate consistency within specific age ranges (see below). One of the studies identified using the inclusion criteria above used the non-modulated version of VBM which reports gray matter density (GMD) as opposed to gray matter volume (GMV) (Mechelli et al., 2004). As it is common to include both modulated and unmodulated samples in VBM meta-analyses (e.g., Linkersdörfer et al., 2012; Barron et al., 2013; Richlan et al., 2013), and it was not an exclusion criteria for our search, we have included it the present analyses. No other whole-brain VBM comparisons in our search reported density as opposed to volume. It is also worth stating that we included García-Pentón et al. (2019), which at the time was a preprint available on *bioRxiv*^4^. The methodology was determined to be of the quality of the other studies found, and as it met all of the inclusion criteria it was determined appropriate to include in the final meta-analyses conducted here.

### Description of Eligible Studies

A total of ten eligible comparisons across eight studies met our inclusion criteria, with a total of sixteen foci for results of bilingual > monolingual contrasts (see Tables 1, 2 for more study characteristics). The number of studies included here is similar to several previous ALE meta-analyses of gray matter morphometry (for example: Rotge et al., 2010; Linkersdörfer et al., 2012; Titova et al., 2013). Foci from monolingual > bilingual contrasts were only reported for one of the eight studies (Olulade et al., 2016), so only the bilingual > monolingual contrast analyses were run and reported here. Out of the ten eligible comparisons, two included separate age groups with unique contrasts eligible for inclusion: older and younger adults (Gold et al., 2013), and adults and children (García-Pentón et al., 2019). The latter study was the only study included that contained children. Of the remaining contrasts, two more consisted of older adults (Abutalebi et al., 2014, 2015), and four in young adults (Mechelli et al., 2004; Ressel et al., 2012; Pliatsikas et al., 2014; Olulade et al., 2016).

**TABLE 1:** Study characteristics.

**Studies**	**N**	**Mean age**		**Sex**			
		**Bilinguals**	**Monolinguals**	**Bilinguals**		**Monolinguals**	
				**Male**	**Female**	**Male**	**Female**
**Children**							
García-Pentón et al. (2019)	28	10.95	10.98	8	6	8	6
**Young adults**							
Ressel et al. (2012)	44	21.5	23.1	11	11	11	11
Pliatsikas et al. (2014)^a^	39	27.5	24.5				
Olulade et al. (2016)	30	22.3	25.9	6	9	8	7
Mechelli et al. (2004)^b^	83						
Gold et al. (2013)	40	31.6	32.2	7	13	8	12
**Older adults**							
Gold et al. (2013)	40	63.9	64.4	10	10	10	10
García-Pentón et al. (2019)	34	69.41	69.29	7	10	7	10
Abutalebi et al. (2015)	38	61.68	60.93	8	11	9	10
Abutalebi et al. (2014)	46	62.17	61.92	9	14	10	13

*^a^Pliatsikas et al. (2014) did not report sex of subjects. ^b^Mechelli et al. (2004) did not report mean age or sex of subjects.*

**TABLE 2:** Number of foci and language status.

**Studies**		**Languages**	
	**Foci**	**Monolingual**	**Bilingual**
**Children**			
García-Pentón et al. (2019)	1	Spanish	Basque + Spanish
**Young adults**			
Ressel et al. (2012)	2	Spanish	Catalan + Spanish
Pliatsikas et al. (2014)	3	English	English + Greek
Olulade et al. (2016)	6	English	English + Spanish
Mechelli et al. (2004)	2	English	English + Italian
Gold et al. (2013)	0	English	English + French, German, Greek, Gujarati, Hindi, Luo, Mandarin, Spanish, Swahili, or Turkish
**Older adults**			
Gold et al. (2013)	0	English	English + Filipino, French, German, Gujarati, Hindi, Igbo, Konkani, Spanish, or Swahili
García-Pentón et al. (2019)	0	Spanish	Basque + Spanish
Abutalebi et al. (2015)	1	Italian	Cantonese + English, Mandarin
Abutalebi et al. (2014)	1	Italian	Cantonese + Mandarin

Methodological variability included software package, where two studies used FSL (Pliatsikas et al., 2014; García-Pentón et al., 2019), and the rest used SPM (Mechelli et al., 2004; Ressel et al., 2012; Gold et al., 2013; Abutalebi et al., 2014, 2015; Olulade et al., 2016). While FSL and VBM are different packages they are both voxel-based morphometric comparisons of gray matter, and studies have been included in the same meta-analysis (for example: Fusar-Poli et al., 2011). Multiple comparison corrections also varied, with FSL-based studies using threshold free cluster enhancement (TFCE; Pliatsikas et al., 2014; García-Pentón et al., 2019), and SPM-based analyses using variable corrections including family wise error (FWE) correction at the voxel level (Mechelli et al., 2004; Ressel et al., 2012; Gold et al., 2013), FWE correction at the cluster level (Abutalebi et al., 2014, 2015), non-stationary cluster correction (Olulade et al., 2016), and some studies additionally reported coordinates for clusters not corrected for multiple comparisons (Mechelli et al., 2004; Ressel et al., 2012). We include all the reported foci in the first analysis (to get a full picture from the limited overall number of eligible studies), and subsequently included only foci corrected for multiple comparisons for a more stringent analysis (described below). It is important to note that Gold et al. (2013) reported no foci for their bilingual vs. monolingual contrast, and Ressel et al. (2012) only reported uncorrected foci. Each of these studies were still included in our foci files for the meta-analyses described below. All eligible contrasts reported their coordinates in MNI space.

### ALE Analysis Methods

All ALE analyses were run using GingerALE 3.0.2^5^ (Eickhoff et al., 2009, 2012; Turkeltaub et al., 2012) using the most recent users’ manual as a guide^6^. The first analysis included all sixteen foci from the ten eligible contrasts, and the second analysis included only foci that had been corrected for multiple comparisons in the original study (thirteen foci from the ten contrasts, subtracting two from Ressel et al., 2012, and one from Mechelli et al., 2004). Next, we ran the same two analyses with the difference of not including the one contrast in children (one foci from García-Pentón et al., 2019). In this case, the first analysis consisted of fifteen foci from the nine total contrasts, and the second analysis (only foci corrected for multiple comparisons) consisting of twelve foci from the nine contrasts (again with two foci removed from Ressel et al., 2012, and one from Mechelli et al., 2004).

GingerALE analyses were carried out in MNI space using the more conservative mask size, and the non-additive ALE method to avoid bias from multiple small clusters that are close together from a single study dominating the results (Turkeltaub et al., 2012). Results thresholds were set using the suggested most conservative and appropriate levels in the GingerALE manual^5^, *p* < 0.001 voxel-wise threshold and *p* < 0.05 FWE cluster corrected threshold with 1,000 permutations. Resulting ALE maps were visualized using Mango software^7^ with the Colin brain template in MNI space^5^.

### Data Availability Statement

In line with transparency of meta-analysis results as suggested in a recent discussion of best practices (Müller et al., 2018), we have provided the datasets for this study including all of our foci files (the input for GingerALE) and all of the GingerALE output files (thresholded maps, maps of foci locations, full descriptions of anatomical locations of resulting clusters, etc.) on our Open Science Framework^8^ project page^9^. These files can be used to rerun our analyses or new analyses if desired.

## Results

### Analysis 1: All Foci, All Contrasts

**FIGURE 1:**
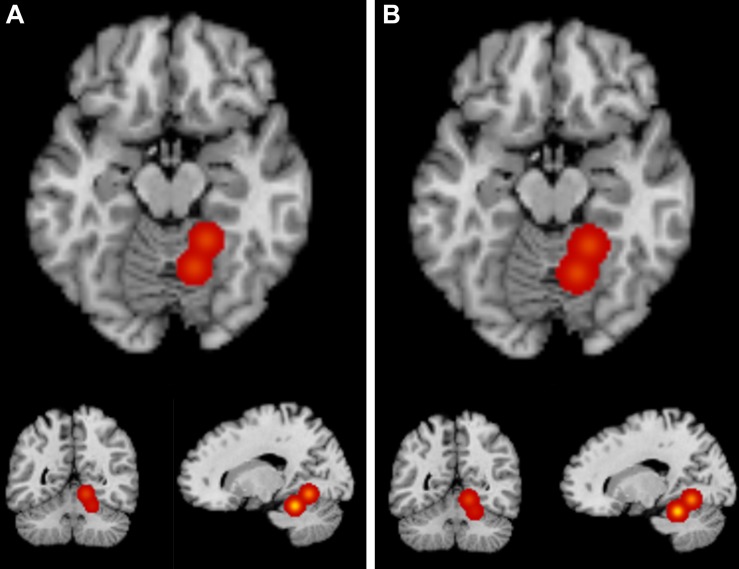
ALE Results. ALE maps from the bilinguals > monolinguals analyses for Analysis 1 **(A)** and Analysis 2 **(B)** at a voxel-wise threshold of *p* < 0.001 and an FWE cluster correction at *p* < 0.05 with 1,000 permutations. Both analyses identified one cluster primarily located in the right culmen within the anterior cerebellum. See Table 3 for cluster information.

For the ALE analysis containing all sixteen foci across ten contrasts, one cluster where bilinguals showed greater GM than monolinguals was identified with two peaks: (18, –44, –20) and (12, –58, –8). This cluster was located primarily in right culmen within the anterior lobe of the cerebellum, extending slightly into the posterior cerebellum and lingual gyrus (BA 19). Foci from Pliatsikas et al. (2014) and García-Pentón et al. (2019) contributed to this cluster. Table 3 contains the peak coordinate information and Figure 1 (left) provides visualization of the cluster.

**TABLE 3:** Cluster coordinates.

		**Anatomical region**	**Peak coordinates**			***P-value***	**Volume (mm^3^)**	***Z***
			***x***	***y***	***z***			
Analysis 1	Peak 1	Right cerebellum, anterior lobe, culmen	18	–44	–20	4.76E-05	13,000	3.9
	Peak 2		12	–58	–8	1.08E-04		3.7
Analysis 2	Peak 1	Right cerebellum, anterior lobe, culmen	18	–44	–20	3.32E-05	16,096	3.99
	Peak 2		12	–58	–8	5.72E-05		3.86

### Analysis 2: All Foci From Contrasts Corrected for Multiple Comparisons

Next, the same analysis was repeated, but removing foci from contrasts that were not corrected for multiple comparisons (as described in section Methods), which included thirteen foci from the ten contrasts. For this ALE analysis, again one cluster where bilinguals showed greater GM than monolinguals was identified with two peaks: (18, –44, –20) and (12, –58, –8). While slightly larger in volume, this cluster is largely identical to the cluster identified in Analysis 1 (primarily in the right culmen within the anterior lobe of the cerebellum, extending into the posterior cerebellum and lingual gyrus-BA 19). Foci from Pliatsikas et al. (2014) and García-Pentón et al. (2019) contributed to this cluster. Table 3 contains the peak coordinate information and Figure 1 (right) provides visualization of the cluster.

### Analysis 3: All Foci, All Adult Contrasts

The results of Analyses 1 and 2 indicated that the right cerebellum showed significant consistency across studies, though only two contrasts contributed to the cluster (Pliatsikas et al., 2014; García-Pentón et al., 2019). As one of the contributing studies represented the only pediatric sample of the included contrasts, it was decided to run the same two analyses without the foci from García-Pentón et al. (2019). The ALE analysis (including fifteen foci across nine contrasts) revealed no significant results at the voxel-wise *p* < 0.001, cluster level FWE corrected *p* < 0.05 threshold. This suggests no consistency across adult VBM studies of GM for bilinguals > monolinguals.

### Analysis 4: All Foci From Contrasts Corrected for Multiple Comparisons for Adult Contrasts

Finally, repeating Analysis 2 for the foci only from adult studies that were corrected for multiple comparisons (twelve foci across nine contrasts), the ALE analysis revealed no significant results at the voxel-wise *p* < 0.001, cluster level FWE corrected *p* < 0.05 threshold. Again, this suggests no consistency across adult VBM studies of GM for bilinguals > monolinguals.

## Discussion

The neuroanatomy of bilingualism continues to be of great interest for the neuroscience of language and in understanding the potential cognitive and brain advantages of learning multiple languages (Baum and Titone, 2014; Li et al., 2014; Bialystok et al., 2016; Grundy et al., 2017). VBM has been a common tool used in studies investigating the neuroanatomy of bilingualism, however, methodology has been quite variable and there is considerable heterogeneity across results. While VBM studies of bilingualism overall have implicated frontal, parietal, and cingulate cortex, along with motor system involvement (e.g., basal ganglia) in aspects of L2 learning and ability (Grundy et al., 2017), very few have directly compared bilingual and monolingual subjects in whole-brain analyses. While correlational and ROI analyses can give important insights into the neuroanatomy of bilingualism, understanding what changes in the brain with acquisition of a second language is a crucial piece of the puzzle that also requires studies using direct comparisons of these groups. Here, we searched the literature for voxel-based morphometry studies that included contrasts that directly compared bilingual and monolingual individuals in a whole-brain analysis. We then ran an ALE meta-analysis on the foci extracted from these studies to probe whether there are consistencies in increased gray matter volume in the brains of bilinguals compared with monolinguals. The initial analysis revealed one cluster primarily in the right anterior cerebellum that was also revealed when only foci from the original studies that were corrected for multiple comparisons were included. However, only two of the ten contrasts contributed to this cluster, including the only study with a pediatric sample (García-Pentón et al., 2019). When the foci from this study were removed, it was revealed that there were no consistencies across the adult VBM studies of bilingualism in GM.

As discussed by Garcia-Penton et al. (2016), the heterogeneity of samples and methodology in the neuroimaging studies of bilingualism has led to a lack of generalization across studies and an overall lack of anatomical support for theories of bilingualism. The studies included in the current meta-analysis highlight these particular sources of heterogeneity in the literature (see Tables 1, 2). While all of these studies include whole-brain VBM comparisons of GM between bilinguals and monolinguals, the samples are all very unique. The languages for the monolingual groups are contained to English, Spanish, and Italian, but the languages of the bilingual groups are unique to each individual study, and in the case of Gold et al. (2013) there are several unique bilingual combinations included. While it is reasonable to expect there will be some anatomical consistencies of bilingualism regardless of the specific languages learned, there may also be unique aspects for certain combinations of languages. For example, bilinguals of two different writing systems (e.g., English and Chinese) may require different levels of cognitive control than bilinguals whose two languages are relatively similar. Antoniou and Wright (2017) discuss how languages that are typologically different may require greater effort to learn, while those that are more typologically similar may require more inhibitory control of overlapping features, thus both leading to increased cognitive reserve via different mechanisms. While there has been little direct testing of this, studies across many bilingual language pairs (such as those discussed here) suggest benefits of various typological similarity. However, more studies aimed at testing this notion directly are needed to further understand the relationship between typological distance and behavior/brain benefits of bilingualism (Antoniou and Wright, 2017).

Other areas of concern for heterogeneity in the current sample of studies is the variability in age of acquisition and proficiency of the bilingual subjects. Not all of the studies included here describe these characteristics fully in their samples. For those that do, how they determined early acquisition is not consistently defined. Age of acquisition can have a significant effect on the anatomy of bilingualism (Berken et al., 2017), which gives caution to the interpretation of the ALE results presented here. Ideally, there would be larger numbers of studies that included lifelong bilinguals and others that include late bilinguals which would allow for parallel analyses. From a methodological point of view, while FSL and SPM versions of VBM both allow for similar analysis there is evidence that suggests results can differ based on the software package used (Rajagopalan et al., 2014; Rajagopalan and Pioro, 2015). Additionally, thresholds varied across studies (as discussed in section Methods) which leaves open the possibility that changing this parameter could lead to different results. With this in mind, the studies included here have relatively similar sample sizes and methodological rigor, making it impertinent to hold one individual study’s results above another. Truly, additional studies across these criteria are needed to fully understand the complex phenomena at play.

It is worth discussing the potential role of the cerebellum, as this is the only consistent result from the ALE analysis. The structure and function of the cerebellum has been linked with verbal language fluency in the general population (Desmond and Fiez, 1998; Richardson and Price, 2009). Clinically, cerebellum lesions have been linked with aphasia (Marien et al., 2000), reduced GMV in the cerebellum has been implicated in developmental dyslexia (Brambati et al., 2004; Eckert et al., 2005), and increased GMV in right cerebellum following reading intervention may be linked with reading improvement (Krafnick et al., 2011). As such, its potential role in second language learning would not be surprising. Pliatsikas et al. (2014) discusses the role of the cerebellum in second language learning within the context of procedural memory and grammatical processing, and Grogan et al. (2009) found a correlation between cerebellum gray matter and fluency across L1 and L2. The average age of acquisition for subjects in these two studies was after the start of formal schooling (Grogan et al., 2009; Pliatsikas et al., 2014), and it is possible that age of acquisition could impact the cerebellum’s role in L2 acquisition or proficiency. Regardless, while the role of the cerebellum in bilingualism is potentially interesting, the results of this meta-analysis in support of this should be taken with caution due to the concerns of heterogeneity described above and the loss of this result when the pediatric study is removed from the analysis.

On a related note, it should be mentioned that the lack of consistency here could suggest a lack of evidence for a “bilingual advantage” (at least related to structural changes in the brain), however, we would caution against this interpretation due to the considerations discussed previously and again below. Instead, we echo the call of Garcia-Penton et al. (2016) for the need of studies with increased sample sizes and better subject characteristic and methodological descriptions so that the intricacies of the bilingual brain can be better understood. While correlational and ROI studies can provide valuable information, whole-brain comparisons in studies with large sample sizes can provide better estimates of group differences between bilinguals and monolinguals. With clear and motivated definitions of early vs. late age of acquisition and fluency in L1 and L2, future meta-analyses of this literature that can utilize a larger literature in more defined areas will provide more specific knowledge related to a “bilingual advantage” for brain structure. Another important consideration for studying the neuroanatomy of bilingualism is utilizing more fine-grained anatomical analyses than VBM. Volume (the most commonly reported metric of the VBM studies included in the present meta-analysis) is the combination of cortical thickness and surface area, and as these measures have unique genetic influence (Panizzon et al., 2009; Winkler et al., 2010), studying these measures may provide more useful information than volume or density alone. Recent studies have begun to investigate bilingualism using cortical thickness (Klein et al., 2014; Archila-Suerte et al., 2018) and surface area (Archila-Suerte et al., 2018; Hämäläinen et al., 2018) providing evidence that these techniques can be employed within the bilingualism field with success. A final consideration for the field as a whole is to engage in more pediatric samples that directly compare bilingual and monolingual children, preferably with a longitudinal component. To fully understand the changes in the brain in acquiring a second language, looking across the lifespan and using longitudinal approaches have tremendous potential to provide valuable information.

Overall, the results of the ALE meta-analyses described here suggest a general lack of consistency in the VBM literature of bilingualism, and highlight the need for larger and more well-defined studies in order to determine the changes that occur in the brain with acquisition of a second language. While these results should be taken with caution due to the overall small number of studies that have employed a whole-brain comparison of bilinguals and monolinguals, this only further indicates the need to design and implement studies that have greater potential to inform the theories and models of bilingual advantage in a meaningful way.

## Ethics Statement

The studies involving human participants were reviewed and approved by Dominican University IRB. Written informed consent from the participants’ legal guardian/next of kin was not required to participate in this study in accordance with the national legislation and the institutional requirements.

## Author Contributions

AK and AD developed the idea for the meta-analysis, executed the literature search, and wrote the manuscript. AD ran the analyses in GingerALE.

## Conflict of Interest

The authors declare that the research was conducted in the absence of any commercial or financial relationships that could be construed as a potential conflict of interest.

## Footnotes

1 https://fsl.fmrib.ox.ac.uk/fsl/fslwiki

2 https://www.fil.ion.ucl.ac.uk/spm/

3 www.ncbi.nlm.nih.gov/pubmed/

4 https://www.biorxiv.org/

5 http://brainmap.org/ale/

6 http://brainmap.org/ale/manual.pdf

7 http://rii.uthscsa.edu/mango/

8 http://brainmap.org/ale

9 https://osf.io/evs4f/
